# Alterations in Cortisol Profiles among Mothers of Children with ASD Related to Poor Child Sleep Quality

**DOI:** 10.3390/healthcare10040666

**Published:** 2022-04-01

**Authors:** Wasmiah Bin Eid, Mengyu Lim, Giulio Gabrieli, Melanie Kölbel, Elizabeth Halstead, Gianluca Esposito, Dagmara Dimitriou

**Affiliations:** 1 Sleep Education and Research Laboratory, UCL Institute of Education, London WC1H 0AA, UK; wasminah.bineid.14@alumni.ucl.ac.uk (W.B.E.); melanie.koelbel.15@ucl.ac.uk (M.K.); l.halstead@ucl.ac.uk (E.H.); 2 Psychology Program, School of Social Sciences, Nanyang Technological University, 48 Nanyang Avenue, Singapore 639818, Singapore; mengyu001@e.ntu.edu.sg (M.L.); giulio001@e.ntu.edu.sg (G.G.); 3 Department of Developmental Neurosciences Unit, UCL Great Ormond Street Institute of Child Health, London WC1N 1EH, UK; 4 Affiliative Behaviour and Physiology Lab, Department of Psychology and Cognitive Science, University of Trento, Corso Bettini, 84, I-38068 Trento, Italy; gianluca.esposito@unitn.it

**Keywords:** autism, ASD, sleep, maternal sleep, child sleep, cortisol

## Abstract

Caregivers of children with autism spectrum disorder (ASD) experience poorer sleep, but studies have not yet used objective measures to investigate how child and caregiver sleep affect each other. In this study, 29 mothers and their child with ASD aged between 6 and 16 years were recruited. Questionnaires measuring child autism, maternal depression, and maternal and child sleep quality were administered. Cortisol salivary samples were also obtained from the mothers over the course of a day. Results revealed that maternal depression is significantly correlated with their subjective sleep quality, sleep latency and daytime dysfunction. Child sleep quality was also found to be significantly correlated with ASD severity. In terms of maternal cortisol profiles, a significant number of mothers showed a flattened diurnal cortisol expression, and children of mothers with a flattened cortisol profile had significantly more sleep problems. Overall, results suggest that maternal and child sleep are affected by the child’s disability but also are mutually related. Future studies may consider employing measures such as actigraphy or somnography to quantify sleep quality and establish causal pathways between sleep, cortisol expression and caregiver and child outcomes. The present study has clinical implications in examining family sleep when considering treatment for ASD.

## 1. Introduction

Developmental disabilities are defined as lifelong conditions that affect an individual’s physical, learning, or behavioural functioning from the early stages of life [1,2]. The category of developmental disabilities is wide-ranging, including neurodevelopmental disorders as well as sensory disabilities, such as hearing loss and blindness [3,4]. Of the neurodevelopmental disorders, Autism Spectrum Disorder (ASD) has received much attention from the scientific community due to the past misattribution of ASD being due to poor parental care [5,6]. However, due to the bi-directional interactions between caregiver and child [7], the truth is more complex. For children with ASD, the added disability of the child often contributes to a vicious cycle of poorer parenting and worse outcomes for both caregiver and child in the long-term [8]. In fact, the negative physical and psychological effects of caring for a child with developmental disabilities and indeed ASD is widely documented [9,10,11,12]. For example, the correlation between parenting stress and problematic child behavior among children with ASD has been established [13]. Additionally, these caregivers are more susceptible to common illnesses such as coughs and colds, as well as mild aches and pains that drive them to visit doctors more frequently [14,15,16,17]. On a physiological level, it appears that slight changes in endocrine and immune processes may be a mediator of poorer physical health among caregivers of children with ASD [15,18,19,20,21].

One area of daily life where the psychological burden of caregiving for a child with developmental disabilities may manifest is in sleep difficulties. Sleep disturbances are also a key marker of endocrine and immune function, and are predictive of increased risk of health issues [22,23,24,25], which may be a potential mediation pathway between caring for a child with ASD and subsequent physical health problems. A commonly used self-report questionnaire used to study sleep in caregivers of children with ASD is the Pittsburgh Sleep Quality Index (PSQI). Findings using the PSQI revealed that caregivers of children with various developmental disabilities (of which ASD comprised more than half of the sample) faced more problems along all sleep parameters, such as sleep latency (i.e., take longer to fall asleep), efficiency, duration and quality, when compared to age-matched controls [21]. These caregivers were also more likely to be categorized as poor sleepers using clinical cut-off scores [21]. In another study using PSQI by [26], caregivers of children with developmental disabilities (including ASD) were found to have longer sleep latency but shorter sleep duration. This finding was also corroborated by studies comparing sleep patterns of caregivers of children with developmental disabilities with population normative data and recommended sleep guidelines [27,28]. Finally, in addition to comparative studies, qualitative interviews have also revealed that caregivers found sleep issues to be one of the most demanding problems when caring for a child with ASD [14,29,30].

Problems with sleep are also common among children with ASD. A review by [31] found that up to 80% of children with ASD, regardless of severity, face some form of sleep problem, and may contribute to worse behavioural problems in the daytime. In fact, a multidimensional study employing polysomnographs, sleep diaries and questionnaire measures similarly revealed that ‘good’ sleepers with ASD have better social interactions and fewer affective issues than ‘poor’ sleepers with ASD [32]. This finding is corroborated by similar studies employing questionnaire-only data [33]. The prevalence of sleep issues among children with ASD warrants treatment guidelines for sleep [34]; however, literature on its clinical implications is still sparse. School-going children aged 6 to 16 years old were of particular interest in this study due to the marked difference in their lifestyle and daily schedules as compared to younger (preschool-aged) children. During this stage in life, children’s schedules are largely dictated by national education policies and their time spent in schools, which typically begin between 8:20 a.m. and 9:00 a.m. in the UK. Schooling therefore necessitates more regular sleep schedules, which tends to diminish the prevalence of sleep problems among typically developing children in this age group [35]. It is then imperative to investigate if these changes are similarly present in the population of children with ASD, or if sleep problems are in fact exacerbated for this group of children.

Furthermore, even though there is extensive literature detailing the effect of caregiving of a child with ASD, to date, studies exploring these sleep disturbances in caregivers of children with ASD have relied largely on subjective measures (e.g., sleep diaries, self-report questionnaires). As subjective measures are prone to biases and lapses in reporting, there is a need for more objective measures of caregiver wellbeing and sleep quality. Together with the findings relating caregivers’ physical and psychological health with physiological changes in endocrine processes [15,18,19,20,21], a potential method of investigation is by using cortisol as biomarker of caregiver stress and sleep. Cortisol secretion follows a diurnal pattern that is negatively affected by sleep problems such as short sleep duration. Cortisol disruption is found to correlate with poorer cognitive functioning [36,37]. Normal cortisol expression on the other hand is found to correlate with improved working memory, processing speed and executive functioning [38].

In autism research, cortisol has been commonly studied in relation to caregiving stress. One such longitudinal study investigating cortisol and stress among caregivers of children with ASD is by [20]. In this study, mothers’ salivary cortisol levels were analyzed together with their children’s behaviour problems as measured by the Scales of Independent Behaviour-Revised (SIB-R). Ref. [20] suggested that mothers caring for a child with ASD are under chronic stress and experience hypoactivity in the hypothalamus pituitary adrenal (HPA) axis, resulting in lower morning cortisol secretion and a blunted cortisol profile compared to mothers caring for a child without a disability. However, few studies have investigated the relationship of sleep and cortisol profiles of caregivers of children with ASD.

Overall, there is a need to understand how we may protect the vulnerable population of children with ASD [39] by also empowering the main stakeholders in caring for these children. Within the context of sleep, given that both the child and caregiver commonly reside in the same household, it is pertinent to study sleep in both child and caregiver as they relate to each other. To our knowledge, there have been no studies to date that aim to study both parent and child sleep simultaneously within the context of ASD. The aim of this study therefore is to assess sleep problems, using both subjective (i.e., self-reported questionnaire) and objective (i.e., cortisol) measures, in mothers as the main caregivers of children with ASD and the children themselves. Additionally, the study aims to evaluate if sleep problems faced by these mothers are related to their mental health outcomes in terms of depressive symptoms, as well as whether the sleep quality of their child has an effect on maternal diurnal cortisol patterns. It is hypothesised that maternal mental wellbeing will be negatively related to the prevalence and severity of their sleep problems.

## 2. Results

### 2.1. Participant Characteristics

Twenty-nine mothers were recruited and eligible for the analysis from the community in London. The mean age of the mothers was 42 years (SD = 7.72, range = 26–61 years-old). The mean age of their children was 10 years (SD = 3.41, range= 6–18 years-old, 5 female and 24 male). There were 21 school aged children (mean age = 9.29, SD = 2.26, range = 6–13 years-old) and 8 adolescent children (mean age = 15.25, SD = 1.58, range = 14–18 years-old). The heritage distribution included 67% White, 28% Asian and 5% Black. All mothers identified themselves as middle class, with 14% working full-time (*n* = 4), 35% working part-time (*n* = 10) but the majority identified themselves as stay-at-home mums (*n* = 15). According to mothers’ Major Depression Inventory (MDI; Ref. [40]) scores, almost half of the mothers experienced depressive symptoms, with 35% of respondents falling into the moderate to severe depression categories (mean = 18.72; SD = 13.79). Children had a clinical diagnosis of ASD carried out by the UK healthcare professionals. In the current study, their mean Childhood Autism Rating Scale second edition (CARS-2 [41]) score was 39 (SD = 5.23; range = 30 to 51.50).

### 2.2. Subjective Sleep Profiles of Mothers

Mothers’ subjective sleep was assessed using the Pittsburgh Sleep Quality Index (PSQI; Ref. [42]). The mean score is 8.14 (SD = 4, range = 2–16). The majority (55.2%) reported experiencing bad sleep quality, with 69% having sleep disturbances, 58.6% taking longer to fall asleep and 41.3% feeling that they do not sleep enough at night (Table 1). Interestingly, 82.8% experienced little to no daytime dysfunction. Daytime sleepiness, as assessed with the Epworth Sleepiness Scale (ESS, Ref. [43]), showed that 24.1% (*n* = 7) of mothers felt moderate to severe excessive daytime sleepiness. After controlling for maternal age and their child’s CARS-2 and Child’s Sleep Habits Questionnaire (CSHQ; Ref. [44]) score, significant correlations were observed for mothers’ MDI with PSQI overall score (*r*(24) = 0.53, *p* = 0.005), PSQI subjective sleep quality (*r*(24) = 0.48, *p* = 0.014), PSQI sleep latency (*r*(24) = 0.49, *p* = 0.011), and PSQI daytime dysfunction (*r*(24) = 0.59, *p* = 0.001).

### 2.3. Subjective Sleep Profiles of Children with ASD

Subjective sleep of children with ASD was assessed using the CSHQ [44] as a gold standard measure. CHSQ data were categorised as per guideline scores. The CSHQ mean score was 52.48 (SD = 10.03, range = 35–73). Accordingly, most children experienced a paediatric sleep disorder when comparing this sample to the clinical norms for CSHQ subscales (86.2% or CSHQ score > 41), with only 13.8% having no or little problems sleeping (*n* = 4). The most prevalent were sleep onset difficulties (72.4%, *n* = 21), sleep duration (65.5%, *n* = 19), daytime sleepiness (65.5%, *n* = 19) and signs of sleep disordered breathing (58.6%, *n* = 17). Moreover, children experienced sleep anxiety (48.2%, *n* = 14), bedtime resistance (44.8%, *n* = 13), night awakenings (37.9%, *n* = 11) and 6.8% (*n* = 2) showed clinical signs of parasomnias. Table 2 reports the precise mean, standard deviation and 95% confidence interval of data from each CSHQ subscale with relation to the possible maximum and minimum scores of each subscale.

Significant positive correlation, after controlling for child gender and age, was observed for CARS-2 score and CSHQ night awakening (*r*(25) = 0.39, *p* = 0.046).

### 2.4. Mothers’ Cortisol Profiles

Cortisol samples were collected from all mothers participating in the study; however, one mother showed unusually high levels of cortisol, and this sample was taken out from eventual group analysis. Mothers’ average morning cortisol was 5.43 ng/mL (SD = 5.25, range = 0.57–22.82), with an average diurnal cortisol ratio of 0.54 (SD = 0.51, range = 0.03–2.21). Precise mean values, standard deviations, ranges and 95% confidence intervals of each time point and overall average daily cortisol levels are reported in Table 3. Normalised cortisol values refer to the percentage of the afternoon or bedtime value with respect to the morning value. AUC^i^ refers to cortisol change (i.e., increase or decrease) while AUC^g^ refers to the area under the cortisol change curve with respect to ground.

**Figure 1:**
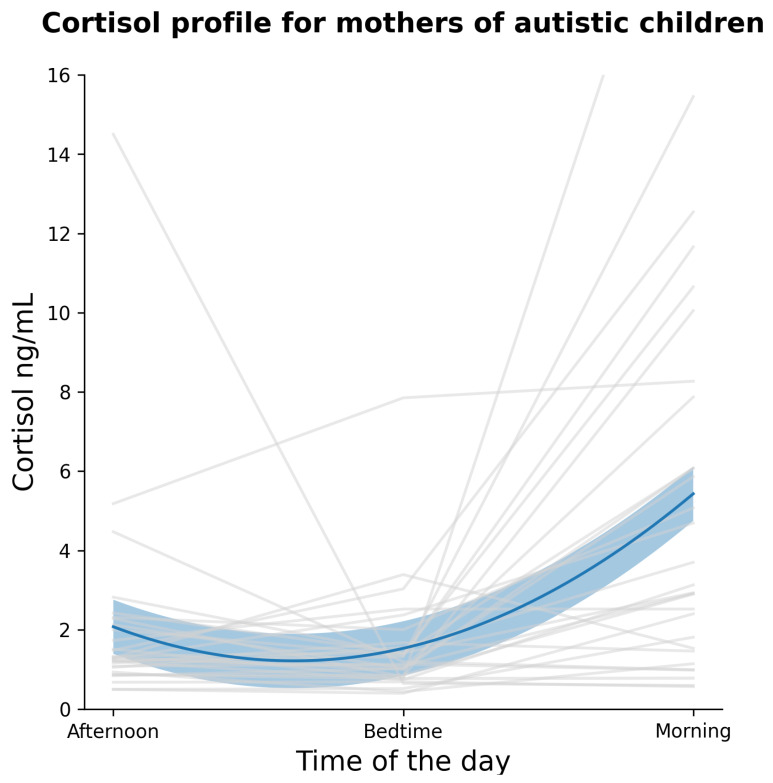
Individual cortisol levels using three data points from morning to evening. Typically, a ‘U’ shape is expected to be seen in healthy cortisol expression. There should be a decrease in cortisol in the evening with an increase in the morning.

It is observed here that there is large variability in cortisol expression across individuals, although it should also be noted that there is no standard reporting of cortisol as well as age related values. Diurnal cortisol profiles are shown in Figure 1.

A significant correlation was observed for MDI (*r*(29) = 0.46, *p* = 0.011) and morning cortisol. However, this correlation reached non-significance after controlling for age.

Children of mothers with a flattened cortisol profile had significantly higher scores on the CSHQ (mean = 55.67, SD = 10.30) compared to children of mothers with a normal cortisol profile (mean = 47.27, SD = 7.31; *p* = 0.017, *d* = 0.90). Differences remained after controlling for the child’s sex, age and CARS-2 score (F(1,24) = 4.86, *p* = 0.037).

## 3. Discussion

To reiterate the aims of this study, we have assessed sleep problems among mothers and their children with ASD using a combination of questionnaire and cortisol measures. Additionally, this study aimed to evaluate if maternal sleep problems are related to their psychological wellbeing, operationalised in this study as MDI scores. It was hypothesised that maternal MDI is related to maternal sleep problems. Finally, this study evaluated if the child’s quality of sleep had an effect on maternal diurnal cortisol expression.

Firstly, by briefly examining the sleep profiles of mothers in the current study, more than half of the maternal sample reported suffering from poor sleep quality, with 69% experiencing sleep disturbances and 58.6% experiencing long sleep onset. These findings are in line with the previous studies showing high PSQI scores in mothers caring for children with developmental disabilities [21,27,28,29]. From the ESS questionnaire, it was revealed that 24.1% of mothers reported moderate to severe excessive daytime sleepiness. It is well established that daytime sleepiness can be classified as a sleep disorder; however, it is not known if the mothers in this study would have been diagnosed clinically with daytime sleepiness. In fact, previous studies have already established that daytime sleepiness is a consequence of sleep deprivation, which also alters sleep architecture. Due to the chronic nature of ASD and long-term caregiving, mothers participating in this study most likely experience prolonged and chronic sleep deprivation. Clinicians and family support workers may pay attention to the needs of caregivers in terms of their sleep quality in an effort to provide more comprehensive care. It is thus possible that their sleep stages have been negatively impacted by having shorter periods of N2 stage [45], which resulted in excessive daytime sleepiness, although this claim could be further demonstrated in future studies using neuroimaging methods such as electroencephalogram (EEG).

Secondly, from the sleep profiles of children with ASD in this study, our data appear to be in line with previous studies. From this sample, a very high prevalence rate of sleep problems was observed, with 86.2% of children scoring within the range of paediatric sleep disorder, and only four children had little or no sleep problems. In comparison to previous studies, the current findings stand at the higher end of prevalence of sleep problems. For example, in [46], sleep problems were recorded in 73% of children with ASD. In another study by [47], of 303 children with ASD aged 2–5 (averaging 3.5-year-olds), only 53% scored high on sleep problems such as sleep onset delay and frequent night wakings. As the aforementioned study had a younger group of children, it is thus plausible to suggest that sleep problems persist and worsen with age in children with ASD. This is also in line with a study of adults with ASD who recorded a high rate of sleep problems and disorders [48]. However, this is one of few studies to report high prevalence of sleep disordered breathing (58.6%), which is previously under-detected in other related studies. From past literature, the most commonly reported the following sleep problems are: bedtime related resistance (e.g., [49,50], sleep onset delay (e.g., [49,50,51,52,53]), frequent wakings and parasomnias such as nightmares (e.g., [50,51,52,53,54]), reduced sleep duration and early morning waking (e.g., [53,55,56,57]). Only one other paper specifically mentioned sleep disordered breathing, revealing that sleep disordered breathing in a sample of 80 children with ASD, aged between 4 and 15 years, was predictive of their stereotyped behavior, social interaction problems, and overall ASD symptoms [58]. Future studies may wish to validate this finding and expand on current understanding of ASD and sleep disordered breathing.

Additionally, it was found that ASD severity was significantly positively correlated with more frequent night awakenings, and that this effect persisted after controlling for child gender and age. This finding aligns with several other studies that also found similar problems of night wakings [47,52,57,59], demonstrating quantitatively and qualitatively different sleep from typically developing children [53]. The reasons for this phenomenon are still debated, with [57] pointing to potential arousal and anxiety factors, with the later [59,60] integrating these factors into a biopsychosocial approach. Symptoms of sensitivity and hyperarousal from these co-morbidities may make it harder for children with ASD to maintain good quality sleep [61,62]. Unfortunately, it is unknown if the present sample of children had other, perhaps undiagnosed, co-morbidities in addition to ASD. As suggested by [59], physiological studies that can provide causal data would be helpful in contributing to the biopsychosocial perspective. Taken together, the relationships between ASD, sleep disordered breathing and night awakenings may point towards a need for more specific ASD treatments and interventions that target nighttime routines, sleep hygiene and sleep habits.

Regarding the relationship between maternal sleep and psychological wellness, findings have supported the hypothesis that more frequent sleep problems are significantly positively correlated to a greater severity of depressive symptoms, particularly in the areas of overall sleep, subjective sleep quality, sleep latency and daytime dysfunction. These effects persisted even after controlling for maternal age, the child’s ASD severity measured by CARS-2 and the child’s sleep quality measured by CSHQ. These findings add more evidence in support of several previous studies that suggested that mothers of children with ASD are chronically sleep deprived and have higher rates of poor psychological function [63,64], as well as depression [65]. The mothers in this sample appear to have undiagnosed sleep onset insomnia, when considering this data within the sleep disorders criteria, although this claim is yet to be verified by professional health practitioners. Nonetheless, the present findings have elucidated the relationship between sleep and mental health within the context of caring for a child with ASD, which gives renewed impetus for healthcare professionals to consider more comprehensive support to caregivers in terms of their psychological needs and to screen for potential mental health issues among parents of children with ASD.

The current study reports maternal cortisol data to examine sleep quality in mothers and how it may be affected by their children’s sleep, which may serve as an initial example and validation of using cortisol as an objective measure of sleep quality in the context of caregiving and ASD, in addition to its more common function of assessing parenting stress. As seen from the large standard deviations and ranges in cortisol data (Table 3), maternal cortisol secretion showed large variations across individuals. However, whether such variability is normal or non-normal for this sample cannot be determined, as norms for cortisol levels are not yet established. Hence, the more relevant indicator of pattern of healthy diurnal cortisol secretion—following a circadian variation of a ‘U’ shape from morning to night—was examined. From this perspective, the current data showed a worrying flattened profile of cortisol in a significant number of mothers. Alterations in cortisol patterns in this manner may be due to chronic sleep deprivation, depression, and other medical conditions. However, the presence of medical conditions here is unlikely as none of the mothers reported any medical conditions, and instead points to the potential effects of sleep problems and poor mental health on cortisol expression. From regression analysis, flattened levels of morning cortisol is correlated with depression, even though the result was not significant after controlling for maternal age. Therefore, it is still inconclusive if cortisol expression in mothers has a definitive impact on maternal mental health. On the other hand, a flattened cortisol profile from mothers was correlated with worse child sleep habits as measured by CSHQ, and this effect persisted even after controlling for the child’s sex, age, and child ASD severity. This finding is supported by numerous studies that found caregivers of children with ASD suffer from worse sleep [14,26,28,60]. The present finding adds to current knowledge by pinpointing that it is the child’s sleep quality that directly correlates with maternal sleep, as the severity of ASD symptoms has been statistically controlled. This suggests that, while maternal sleep quality is influenced by the demands of caregiving, parent and child sleep patterns are also mutually related, independently of the child’s health conditions. In fact, mothers of typically developing children who have sleep problems have poor sleep quality themselves [65]. A child’s sleep problems have an impact not only on maternal sleep health, but also overall family functioning and sleep quality of other family members [66], parenting quality and even marital relationship [50]. Therefore, it is of importance that sleep is investigated in relation to other members of a household. Of course, as previously mentioned, it is also important to make use of these findings when considering interventions that would be appropriate for the family, taking into account the bidirectional influence of sleep quality and overall health and functioning of the household caring for a child with ASD.

### Limitations and Future Studies

In addition to the suggested study expansions described in the discussion above, this study is not without its limitations. While this study has methodological strengths such as the use of objective assessments of cortisol analyses and simultaneous sleep measures for both mother and child, this study was limited by its small sample size and cross-sectional design. It remains to be seen if the findings can be generalised to other caregivers in different countries and social support systems, or if the present findings are sufficiently specific, given that the age group of children included in this study is rather large (i.e., ages 6 to 16). Related studies have found developmental trajectories in sleep among children aged 7 to 19 and 5 to 14, respectively, with a general decrease in sleep problems for typically developing children as they progress to adolescence [67,68]. Similarly, Ref. [69] also found that a large proportion of children had declining sleep problems as they grew from ages 5 to 17 (i.e., only 8% experienced persistent sleep problems). It remains to be seen if similar trajectories are observed for children with ASD. Therefore, to provide a more developmental perspective within the context of ASD, future studies may consider further segmenting the age group into two categories: school-going children (aged 6 to 13; approximately Years 7, 8, 9 in the UK education system) and adolescents (aged 14 to 16; approximately Years 10 and 11 reflecting preparation for the national GCSE examinations). Nonetheless, the methodology used here may be sufficiently rigorous that future studies could use a similar experimental design to replicate the current findings with a larger sample of participants. However, additional factors such as lifestyle factors and family structure (e.g., presence of father, other prominent caregivers, siblings) ought to be considered as potential covariates. It may also be interesting to consider child mental health and wellbeing together with mother’s depression to obtain a more comprehensive perspective of mother-child functioning. Finally, despite the addition of cortisol as an objective measure, self-reported questionnaires are still subject to a range of biases. Particularly in this context, child measures on the CSHQ and CARS-2 that were answered by their mothers may be susceptible to social desirability bias, particularly if they are caregivers who show high levels of defensiveness to minimize instances of problems of stress between themselves and their children [70]. To address this issue, the Marlowe–Crowne Social Desirability Scale (SDS; Ref. [71]) or its shortened version [72] could be implemented alongside the other questionnaire measures. The problem of self-report biases in questionnaires could also be addressed in future studies by implementing other objective measures of sleep, such as somnography or actigraphy, as well as portable neuroimaging methods to detect changes in brain activation during rapid eye movement (REM) and non-REM sleep. Based on the latest methodological advancements such as hyperscanning in neuroimaging [73,74] and initial findings of mother-child synchronicity and sleep [75], these objective measures can be used in future studies to investigate interpersonal phenomena and the co-regulation of neurological, physiological, behavioral processes in naturalistic settings between the mother and child during sleep.

Overall, the flattened cortisol expression patterns reported here have clear implications for mothers’ quality of health. Chronic flattened cortisol secretion may be indicative of physical symptoms such as weakness, fatigue, and low blood pressure, with the possibility of developing more serious medical conditions such as Addison’s disease in the future. Taken together, future studies ought to examine sleep architecture and cortisol to establish a clear causal pathway, such that potential treatments, interventions or appropriate support for both mother and child can be formulated.

## 4. Materials and Methods

The study was conducted in accordance with the Declaration of Helsinki and approved by the UCL Research Ethics Committee (approval number 16682/001). Data are not publicly accessible but can be made available on request to D.D.

### 4.1. Participants

A total of 29 mothers of children diagnosed with ASD, together with their child (*N* = 58) were recruited. All parents were recruited from the London area through online advertisement and autism-related social events and workshops held by the SERL lab using snowball sampling methods [76]. Inclusion criteria included mothers who are aged above 18 years and who are currently caring for a child aged between 6 and 16 years who is clinically diagnosed with ASD (i.e., diagnosed by a general practitioner, paediatrician or other health professional). The child must be living habitually with their mother at home. Exclusion criteria included mothers who are also caring for another person (e.g., child, parent, partner, other relative, or friend) with a chronic illness or other children with other developmental disorders. Additionally, mothers who were experiencing chronic stressors such as divorce or bereavement within the last 12 months were also excluded from the study.

### 4.2. Questionnaires

In addition to the CARS-2 [41], MDI [40], PSQI [42], ESS [43] and CSHQ [44] described below, demographic information on participants’ heritage, age and sex were also collected from both mother and child. Mothers were also asked to report their current employment position and social class (e.g., lower-class, middle-class, upper-class) as a measure of socioeconomic status.

#### 4.2.1. Childhood Autism Rating Scale-Second Edition (CARS-2)

Consisting of 15 items rated on a 4 point scale (ranging from 1 = age-appropriate behaviour to 4 = atypical behaviour), CARS-2 [41] is a behavioural rating scale that measures ASD severity. Questions in CARS-2 focus on interpersonal relations, the use of body and objects, affective response, imitation, adjustment to change, and visual and listening response. Total scores range from 15 to 60, where the lowest score range (15–30) indicate no or minimal signs of autistic behaviour. The categories are followed by mild-to-moderate signs of autistic behaviour (30–36) with the highest range (37–60) indicating strong signs of autistic behaviour.

#### 4.2.2. Major Depression Inventory (MDI)

As one of the most widely used questionnaires measuring severity of depressive symptoms, MDI [40] is a 10-item self-rated scale ranging from 0 (at no time) to 5 (all of the time), reflecting how much of the time during the last two weeks individuals have experienced various depressive symptoms. For example, items include feeling in low spirits or sad, or losing interest in day-to-day activities. Scores are then summed to yield a score of depression, where a higher total score indicates greater depressive symptoms. The categories are: no or doubtful depression (0–20), mild depression (21–25), moderate depression (26–30) and severe depression (31–59). A clinical cut-off score of 26 was recommended to distinguish between depressed and non-depressed individuals with a sensitivity of 0.86 and specificity of 0.82 [40].

#### 4.2.3. Pittsburgh Sleep Quality Index (PSQI)

PSQI [42] is a 24-item self-rated questionnaire assessing sleep quality and sleep disturbances over the previous month. Items such as “have to get up to use the bathroom”, “cannot breathe comfortably” and “had bad dreams” are rated on a four-point scale, depending on the frequency of occurrence (0 = not during the past month, 1 = less than once a week, 2 = once or twice a week, 3 = three or more times a week). PSQI can be scored according to seven subscales: subjective sleep quality, sleep latency, sleep duration, habitual sleep efficiency, sleep disturbances, use of sleeping medication, and daytime dysfunction. Additionally, a global score from the sum of the subscales can be derived, ranging from 0 to 21. Higher scores indicate increased sleep problems. Cronbach’s alphas for each subscale range from 0.35 for sleep disturbances to 0.76 for habitual sleep efficiency and subjective sleep quality, while the Cronbach’s alpha for the global score is 0.83. A clinical cut-off total score of 5 was able to correctly distinguish between 88.5% of patients with sleep disorders and controls.

#### 4.2.4. Epworth Sleepiness Scale (ESS)

ESS [43] is a questionnaire measuring general daytime sleepiness on a scale of 0 (would never doze) to 3 (high chance of dozing). Items measured the likelihood of respondents either dozing or falling asleep in eight different everyday situations such as sitting and reading, watching TV, and sitting quietly after a lunch without alcohol. ESS total score can reliably distinguish between individuals with no sleep disorders from a range of sleep-related diagnostic groups including obstructive sleep apnoea syndrome, narcolepsy and idiopathic hypersomnia, but not from primary snoring, insomnia or periodic limb movement disorder [43].

#### 4.2.5. Child’s Sleep Habits Questionnaire (CSHQ)

Consisting of 45 items in the past week, CSHQ [44] is a subjective measure of child sleep problems (e.g., sleep anxiety and night wakings) and sleep behaviour (e.g., sleep duration, bedtime and sleep onset behaviour). Items on CHSQ are rated on a 3-point scale: 1 = Rarely (i.e., never or 1 time within the past week), 2 = Sometimes (i.e., 2–4 times within the past week) and 3 = Usually (i.e., 5–7 times within the past week). CSHQ scores can range from 33 to 99 points, and a score above 41 indicates a paediatric sleep disorder.

### 4.3. Cortisol Salivary Samples

Mothers were asked to take three samples of their saliva using salivary collectors labelled with unique ID numbers. The tubes were colour coded for each time point and had the time of expected collection written on the paper: AM- Yellow; PM- Blue; Evening- Green. The first sample was taken no later than 30 min after awakening, followed by one in the afternoon at 4:00 p.m. and finally before habitual sleep. We used an oral fluid collector (OFC) from Soma Bioscience Ltd. (Wallingford, UK) to collect the saliva samples individually. It consists of a synthetic polymer swab designed and mixed with an OFC buffer. The collected sample is unaffected by recent food and drink ingestion and can be stored at room temperature for several weeks. An enzyme immunoassay (EIA) test kit was used (Soma Bioscience Ltd., Wallingford, UK) to determine the salivary assay range for cortisol (0.25–32.0 ng/mL). Cortisol profiles were assessed using all 3 samples, compared to the individual group mean. Values are reported for raw morning cortisol and diurnal ratio (DCR) (PM/AM).

### 4.4. Procedure

Informed consent was obtained from the child and their parent or legal guardian. Upon obtaining informed consent, mothers filled in the questionnaires as listed above and provided their salivary samples at three different time points over the course of a day. Questionnaires and cortisol samples were both collected within a week of obtaining informed consent.

### 4.5. Analytic Plan

Statistical analysis was performed using SPSS^®^ version 26 (IBM Corporation, Armonk, NY, USA) for Mac^®^, while correlational analyses were performed using Python (v. 3.5.8) on Linux (Kernel 5.4.0-73-generic). All results are shown in Mean and SD unless otherwise stated. Data were assessed for normality and homogeneity of variance, using a Shapiro–Wilk test. Parametric or non-parametric tests were then chosen for comparison, where most appropriate. Normative clinical data developed by [44] were used to determine a clinically significant cut-off score for the CSHQ subscales. Any score above the clinical sample mean were defined as having difficulties with their sleep in the following categories: bedtime resistance (>9.43), sleep onset (>1.80), sleep duration (>4.94), sleep anxiety (>7.09), night wakings (>5.69), sleep disordered breathing (>4.71), parasomnias (>11.22) and daytime sleepiness (>11.99).

## 5. Conclusions

In conclusion, this study served as an initial examination into the mutual effects of sleep between mothers and children with ASD, by making use of a combination of subjective and objective measurements. While it is well-established that caregivers of children with developmental disabilities such as ASD often face worse outcomes, this study showed that, beyond simply considering the disability by itself, it is also the bi-directional interactions between mother and child that can contribute to and mutually affect the quality of life between the dyad, particularly in the domain of sleep and mental health. Future research may choose to expand upon the present study by including a more diverse and larger sample size, segmenting the children into finer developmental stages, and also considering the simultaneous application of other modes of measurement such as actigraphy and EEG.

## Figures and Tables

**Table 1:** Pittsburgh Sleep Quality Index Scores by Subscale Use of Sleeping Medication subscale should instead be interpreted along the categories (0) Not during past month, (1) Less than once a week, (2) Once or twice a week, (3) Three or more times a week.

PSQI Subscale	Very Good/No. (%)	Fairly Good/No. (%)	Fairly Bad/No. (%)	Very Bad/No. (%)
Subjective Sleep Quality	6 (20.7)	7 (24.1)	12 (41.4)	4 (13.8)
Sleep Onset Latency	5 (17.2)	7 (24.1)	11 (37.9)	6 (20.7)
Sleep Duration	7 (24.1)	10 (34.5)	7 (24.1)	5 (17.2)
Habitual Sleep Efficiency	16 (55.2)	3 (10.3)	7 (24.1)	3 (10.3)
Sleep Disturbance	0 (0.0)	9 (31.0)	20 (69.0)	0 (0.0)
Use of Sleeping Medication	11 (37.9)	5 (17.2)	1 (3.4)	2 (6.8)
Daytime Dysfunction	16 (55.2)	8 (27.6)	4 (13.8)	1 (3.4)

**Table 2:** Child’s sleep habits questionnaire scores by subscale.

CSHQ Subscale (Theoretical Range)	Mean (SD)	95% Confidence Interval
Bedtime Resistance (6–18)	9.66 (3.35)	[8.38, 10.93]
Daytime Sleepiness (7–24)	13.79 (4.48)	[12.09, 15.50]
Night Wakings (3–9)	4.69 (1.97)	[3.94, 5.44]
Sleep Anxiety (4–12)	6.93 (2.70)	[5.90, 7.96]
Sleep Disordered Breathing (3–14)	7 (3.72)	[5.88, 8.42]
Sleep Duration (4–9)	6.03 (2.03)	[5.26, 6.81]
Sleep Onset (1–3)	2.10 (0.82)	[1.79, 2.42]
Parasomnias (3–11)	5.14 (1.88)	[4.42, 5.85]

**Table 3:** Group cortisol patterns of mothers.

Cortisol Measure	Mean (SD)	Range	95% Confidence Interval
Average Cortisol/nM	2.93 (2.02)	0.65–8.37	[2.14, 3.72]
Morning (within 30 min of waking)/nM	5.60 (5.26)	0.6–22.82	[3.56, 7.64]
Afternoon (4 p.m.)/nM	1.63 (1.08)	0.5–5.2	[1.21, 2.05]
Before Habitual Sleep/nM	1.56 (1.43)	0.4–7.85	[1.00, 2.12]
Normalised Afternoon/%	59.62 (83.35)	6.62–451.52	[27.30, 91.95]
Normalised Bedtime/%	52.84 (51.30)	3.42–221.57	[32.95, 72.73]
AUC^i^	15.63 (10.04)	3.93–36.00	[11.74, 19.53]
AUC^g^	−17.97 (23.26)	−96.99–10.74	[−26.99, −8.95]

## Data Availability

Data are not publicly accessible but can be made available on request to D.D.
